# Integrating Virtual Worlds with Tangible User Interfaces for Teaching Mathematics: A Pilot Study

**DOI:** 10.3390/s16111775

**Published:** 2016-10-25

**Authors:** Graciela Guerrero, Andrés Ayala, Juan Mateu, Laura Casades, Xavier Alamán

**Affiliations:** 1 Departamento de Ciencias de la Computación, Universidad de las Fuerzas Armadas ESPE, Sangolquí 171-5-231B, Ecuador; rgguerrero@espe.edu.ec; 2 Departamento de Ingeniería Informática, Universidad Autónoma de Madrid, Madrid 28049, Spain; cesar.ayala@estudiante.uam.es (A.A.); juan.mateu@estudiante.uam.es (J.M.); 3 Departamento de ciencias, Florida Secundaria High School, Catarroja 46470, Spain; lcasades@florida-uni.es

**Keywords:** virtual worlds, mixed reality, tangible user interfaces, e-learning

## Abstract

This article presents a pilot study of the use of two new tangible interfaces and virtual worlds for teaching geometry in a secondary school. The first tangible device allows the user to control a virtual object in six degrees of freedom. The second tangible device is used to modify virtual objects, changing attributes such as position, size, rotation and color. A pilot study on using these devices was carried out at the “Florida Secundaria” high school. A virtual world was built where students used the tangible interfaces to manipulate geometrical figures in order to learn different geometrical concepts. The pilot experiment results suggest that the use of tangible interfaces and virtual worlds allowed a more meaningful learning (concepts learnt were more durable).

## 1. Introduction

From an early age, manipulating objects has shown a number of advantages for the cognitive development of children. One of the first formalizations of this idea can be found in Piaget [1]. He proposed four states of cognitive development in which the state of “concrete operations” is a preliminary condition for achieving the state of “formal operations”. In the “concrete operations” state logical reasoning is done through objects that are real in the sense that you can see them or manipulate them. This is the concept of constructivism. Papert [2] elaborates this concept through the use of programming environments such as Logo, in order to allow children building their own knowledge through physical representations of abstract concepts.

Fitzmaurice [3] proposed one of the first approaches to tangible interfaces, which he called graspable user interfaces. These allowed direct control of electronic or virtual objects through physical handles. Some years later Ishii and Ullmer [4] defined tangible user interfaces (TUIs) as systems that mix physical with digital information, employing physical artefacts both as representations and as control elements for computational media. Tangible user interfaces are physical objects or artefacts that are electronically augmented in order to trigger digital events [5]. Shaer and Hornecker [6] indicate that tangible interfaces allow to use physical objects to represent and manipulate digital data.

There are some examples in the literature on creating interfaces based on tangible kits in order to help students in the learning process. Most of these tangible kits are made up of: tangible items or artefacts (wooden blocks, LEGO constructions, robots, animated toys, etc.); a specific software (that simulates some situation with educational interest); and some hardware elements that allow one to communicate the tangible elements with the software (camera, sensors, actuators, etc.). Ullmer et al. [7] proposes a classification of tangible interfaces in three types: interactive tables where the tangible objects are manipulated; construction kits based in modules that can be assembled with each other; and token and constraint systems which combine some characteristics of the first two.

Zuckerman [8] proposed a classification of tangible (or manipulative) interfaces into two types: Froebel-inspired manipulatives (FiMs) and Montessori-inspired manipulatives (MiMs). The FiM tangible interfaces use materials that simulate real-world structures, such as wooden blocks that can be used to build a castle, or pieces of plastic that can be used to build an airplane. We can find many educational projects based on FiMs: ActiveCube [9], Topobo [10], Block Jam [11] and roBlocks [12].

On the other hand, Montessori-inspired manipulatives (MiMs) model more abstract structures where each block represents, for example, a mathematical operation or any other abstract concept. SystemBlocks and FlowBlocks [8] are two projects based on MiMs that aim to teach mathematical concepts such as probability, dynamical systems and programming concepts (loops, variables and branching).

Tangible interfaces in education have been applied to various areas, such as literature or storytelling (KidPad [13]), music (Reactable [14] and Block Jam [11]), science and, especially, mathematics and programming (see below). At the same time, most tangible interfaces experiments involve transversal skills such as reading comprehension, as well as oral and written expression.

Many projects have been developed in the field of programming using tangible interfaces. The reason for this is the difficulty that children have while learning the abstract syntax of programming languages. For example, Quetzal [15] is a tangible programming language for controlling Lego Mindstorms robots. In the same line, Tern [15] is another tangible programming language in which you can connect wooden blocks (like puzzle pieces) forming structures that control virtual robots represented on the computer. Similar to Tern, T-Maze [16] allows to build your own program and control a virtual character in order to get it out of a maze. Finally, Sheets [17] allows you to create programs using paper cards that allow step-by-step execution of the program, with the aim to understand how it works.

Tangible interfaces are also used for construction and interaction in 3D environments in a simpler and more intuitive way. For example, projects like ActiveCube [9] or the project proposed by Anderson et al. [18] make use of physical building blocks in order to build virtual structures.

There are some studies in the literature assessing the performance of learning methodologies that use tangible objects. Okun [19] presented a comparative study on the use of Tangram to teach Mathematics, both using wooden blocks and virtual blocks in a computer. It was observed that the use of tangible objects showed more benefits, especially at early ages. Klahr et al. [20] performed an experiment in which children had to build a car using a kit of pieces, trying to optimize its speed. For this experiment there were two student groups: one built a physical car and the other built a virtual car. They concluded that children performed better when using the real, tangible car. Finally, Manches [21] performed an experiment in which children learned to break down numbers using different partitioning strategies. For this experiment there were three student groups: one using a virtual environment, other using tangible objects and last one using paper and pencil. Children got more correct solutions when using the physical option compared with the two other options.

In the projects commented above the tangible interface interacted with ad-hoc software developed specifically for each experiment. This software was not directly reusable in other projects with other tangible interfaces. Our proposal is to use a virtual world platform instead. There are many advantages in this approach: adding new tangible interfaces is easier; the final applications have multiuser capabilities without any extra effort; the application may use additional tools such as chats, asynchronous messaging, etc.; 3D models made with standard tools such as SketchUp or Blender are easily included; and non-technical users may edit or even build the whole virtual world. The objective is to allow teachers with no knowledge of programming to build their own mixed reality educational applications.

In references [22,23], virtual worlds are defined as computer generated environments that use two or three dimensional computer generated images to represent elements of the physical world or imaginary scenarios. In these worlds, users are able to perform a wide range of interactions with the synthetic elements of the virtual world and with other users [24].

The development of virtual world technologies has opened new opportunities in the educational field. According to the U.S. National Science Foundation, virtual worlds have the potential to play a significant role in education [25]. Virtual worlds allow different types of educational activities such as role playing, problem based learning, collaborative simulations (learn by simulation), collaborative construction (building activities), and language learning among others. Collaborative simulations and collaborative construction are the most common activities in virtual worlds [26].

Mixed reality combines physical world elements (tangible interfaces) with virtual worlds [27]. According to Milgram’s taxonomy [28] there are two possible scenarios for mixed reality: real experiments with virtual overlays, which are called augmented reality (AR); and virtual experiments that are complemented with real world components, which are called augmented virtuality (AV). The research described in this paper is in the scope of AV environments, thus the physical component for interaction is a key element: we will call it the tangible interface [29]. In this paper we describe two prototypes of tangible interfaces that have been developed and evaluated in the area of mixed reality in education, using a middleware developed by Mateu [30].

Research done by Starcic et al. [31] describes a system for teaching geometry using tangible interfaces, aimed to inclusive education. This study concludes that tangible user interfaces are an effective approach for teaching geometry particularly for students with motor skill problems.

The research done by Ishii [4] explores the combination of tangible elements with virtual reality, which allows users to understand and manipulate digital information using everyday objects as user interfaces. The aim of tangible interfaces in virtual reality is to close the gap between virtuality and the physical environment. According to Takuya et al. [32], several research groups have studied tangible user interfaces for modelling 3D objects. This review confirmed that this approach allows users to interact using human natural constructionist skills overriding the limitation of traditional user interfaces.

Studies done by Thriona and Klahr [33] indicate that the use of physical objects in education helps the exploration and development of skills in learners. When these tools are combined with objects in virtual reality an improvement in the students’ knowledge acquisition process is noticed.

Kaufmann et al. [34] proposed a virtual model builder that used augmented reality, called Construct3D, which was focused in teaching mathematics and geometry by means of using geometric shapes (lines, planes, cubes, spheres, cylinders and cones). This tool provides basic editing with the support of virtual reality glasses and a pencil to perform movements like selecting figures and operations. After some experiments that involved a group of students, the author concludes that the students presented visual coordination problems whit the system: an accuracy issue was noticed when performing the activities involving interactions on a virtual 3D environment. However, they were highly interested and motivated with the activity.

Wang and Dou [35] designed a tangible interactive tool called StoryCube, which helped children to interact with virtual reality objects through the use of a tangible user interface, replacing the mouse and keyboard. The tangible interface allowed children to operate and modify virtual objects while they narrated a story in a virtual world, allowing them to pick up elements of the story and its characters to decorate their own history. The tangible interface prototype was designed using RFID (Radio Frequency IDentification) tags and readers, accelerometers, buttons and a joystick. After the evaluation of the tangible interface it was observed that children preferred to play with buttons and to manipulate objects, compared to more traditional user interface alternatives. There are other examples of tangible interfaces applied to virtual reality, such as KidPad [36], Telling tales [37], the Tangible Interface for Storytelling TOK [38], StoryRooms [39], ToonTastic [40], PuzzleTale [41] and TellTable [42].

It is important to mention that tangible interfaces in virtual reality contribute to the development of collaborative work CSCW (Computer-Supported Cooperative Work) [43]. An experiment by Gu et al. [44] shows that the mixed reality interaction supports group collaboration when students are challenged on the designing of a common task. There is a wide range of input devices for augmented reality that have been developed for collaborative work such as Towards Natural [45], Built-it [46] and DiamondTouch [47].

Other project using mixed reality in education is the environment proposed by Nikolakis et al. [48]. In this project, students learn geometry by using a haptic glove that allows the creation of geometrical objects. This project was assessed with high school students, concluding that the mixed reality environment provides a more efficient problem solving approach for learning geometry.

In order to be able to manipulate 3D objects naturally, at least six degrees of freedom (6-DOF) are needed, three for translation on the *X*, *Y*, *Z* axis, and three for rotation around them. Zhai [49] shows the difficulties in making a standard 6-DOF device. In first place, the manufacturing costs are higher. In second place, there is only a very limited knowledge about what properties should have a good 6-DOF device in order to make it both ergonomic and functional.

Mine [50] designed an immersion device aimed to allow the user performing tasks within a virtual reality environment through various interaction techniques, including a remote control (a handheld widget), which allow immersion in a virtual space creating a perception of standing in direct interaction with virtual objects. This device was designed for two hands interaction: each hand interacted with a 6-DOF sensor and the user wore a stereoscopic viewer in order to perceive movements in 3D. But the author says he has had problems regarding the use of the device, since the movement was not intuitive for users, possibly due to magnetic interference affecting the sensors. Additionally, the author noted the high hardware cost of the system.

Another similar study is the 3-Draw system developed by Sachs et al. [51], which was designed for using both hands: each hand controls a 6-DOF sensor device in order to interact with images on a conventional screen. Users were able to draw three dimensional contours by combining both devices. Sachs reported that the user interface was natural and fast, and the simultaneous use of the two hands provided a kinaesthetic feedback that allowed users to feel as if they were holding the objects displayed on the screen.

JDCAD is an interactive 3D modelling system designed and built by Liang [52]. It uses a 6-DOF sensor combined with a helmet with an embedded screen. Liang showed promising results when compared to conventional systems, since it was easier to use, and time savings were observed when performing design tasks. A similar project was developed by Shaw and Green [53]: a design system with two 6-DOF tangible interfaces, which were used to draw free form polygonal surfaces in a virtual reality environment. We may cite other works with similar characteristics such as: 3D animations [54], 3DM (three Dimensional Modeller) [55], WIM (world in miniature) system [56] and TwoHanded Interface [57].

The tangible user interface Novint Falcon [58] is a haptic device with USB connection that was designed to replace the mouse. Users are able to control it making movements in three dimensions: (i) up and down; (ii) forward and backward and (iii) from right to left. As the user moves the bubble grip, sensors communicate with the computer. This bubble allows users to manipulate objects in a virtual 3D application, providing tactile feedback.

However, to our knowledge, all the existing projects in this area use ad-hoc 3D applications for the virtual reality part of the system. Therefore, changing the learning application (for example, adding a new activity) cannot be done by end users (teachers), but has to be programmed by the developers. In our proposal, mixed-reality is implemented using a full-fledged virtual world, based in the OpenSim platform (which is an open software version of Second Life). This platform provides editing capabilities to any user, in a quite easy fashion. Therefore, the use of a virtual world platform instead of an ad-hoc 3D application will allow teachers to extend the educational applications, or even to create new ones by themselves. The main objective of this study is to assess whether the use of virtual worlds and tangible interfaces is beneficial for student learning when compared to more traditional forms of learning. This study provides evidence that students using the aforementioned technologies, exhibit increasing concentration and interest int the activities. They also retain knowledge significantly better as they outperformed control group students in a delayed post-test conducted two weeks after the study.

## 2. Materials and Methods

Two devices have been developed to be used as tangible interfaces for the creation of mixed reality educational applications. The first “tangible interface” device is FlyStick, which allows the user to control a virtual object in six degrees of freedom. An educational application for teaching conic sections was developed using this device.

The second “tangible interface” device is PrimBox which allows the user to modify virtual objects, changing attributes such as their position, size, rotation and color. An educational application for teaching the students how to communicate geometrical knowledge using technical language was developed using this device.

The architecture for this mixed reality environment is based on the Virtual Touch middleware developed by Mateu et al. [30], which allows integrating different types of tangible interfaces and different types of hardware technologies with virtual worlds.

Both devices use a set of sensors that communicate with the virtual world through the middleware. The Phidgets technology was chosen for the sensor implementation, because it provides a low cost and reliable hardware, as well as interface libraries for a handful of programming languages.

### 2.1. FlyStick Tangible Interface

As discussed in Section 1, mixed reality has a high potential in the field of education, but has not been widely adopted yet because of the need of more mature technological elements for its deployment in the classroom.

FlyStick is a tangible interface that allows interacting with virtual objects in six degrees of freedom (translation on three mutually perpendicular axes and rotation on each of them). When using a device of this kind for controlling 3D objects some of the difficulties that arise while using 2D interfaces (such as the mouse and touch screens) are avoided. One of the main limitations of 2D devices is that they are unable to control 3D positions and orientations on a straightforward way. These difficulties have been traditionally addressed through the combination of pressing keys and using the mouse (or touch screen) to specify the 3D actions to be done.

The proposed tangible interface was designed taking into account how humans transmit information to a device using their limbs. A human limb can send and receive information through two types of muscle contractions, either by applying force or by performing a displacement. Devices capable of interpreting forces are called isometric devices while those that interpret movements are called isotonic devices.

FlyStick combines isotonic and isometric interactions allowing the translation and rotation of elements in a 3D virtual world. Students can be involved in constructivist educational experiments, in which students may abstract concepts by means of the perception of a direct involvement of their actions in the virtual world. In this research the proposed experiment is related with learning abstract concepts in geometry, specifically the conic sections.

#### 2.1.1. Design of the Virtual World

OpenSim [59] has been chosen as the platform to host the virtual world because, besides being open source and free (which makes it more accessible to schools), it allows the creation of virtual environments and provides user management. Another advantage of OpenSim over other virtual world environments is that it is not subject to the policies and particular interests that very frequently are related to proprietary servers.

A virtual island was created for the implementation of educational activities related to teaching geometry, where students can interact with various virtual objects. The virtual world implemented for this experiment is housed in an OpenSim server. Access to the virtual island is made using browsers compatible with OpenSim protocols. Within it, students can interact with various virtual objects through the tangible interface devices.

**Figure 1:**
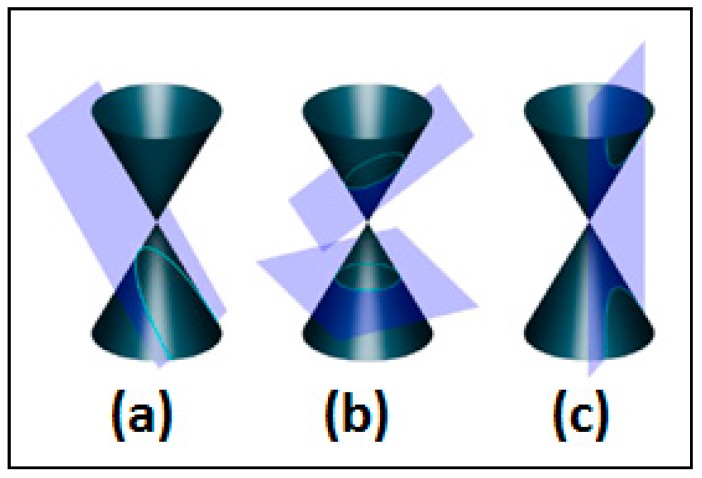
Conic sections: (**a**) parabola; (**b**) circle/ellipse and (**c**) hyperbola.

The activity is focused on teaching conic sections as the curves obtained by intersecting a plane with a cone (Figure 1).

**Figure 2:**
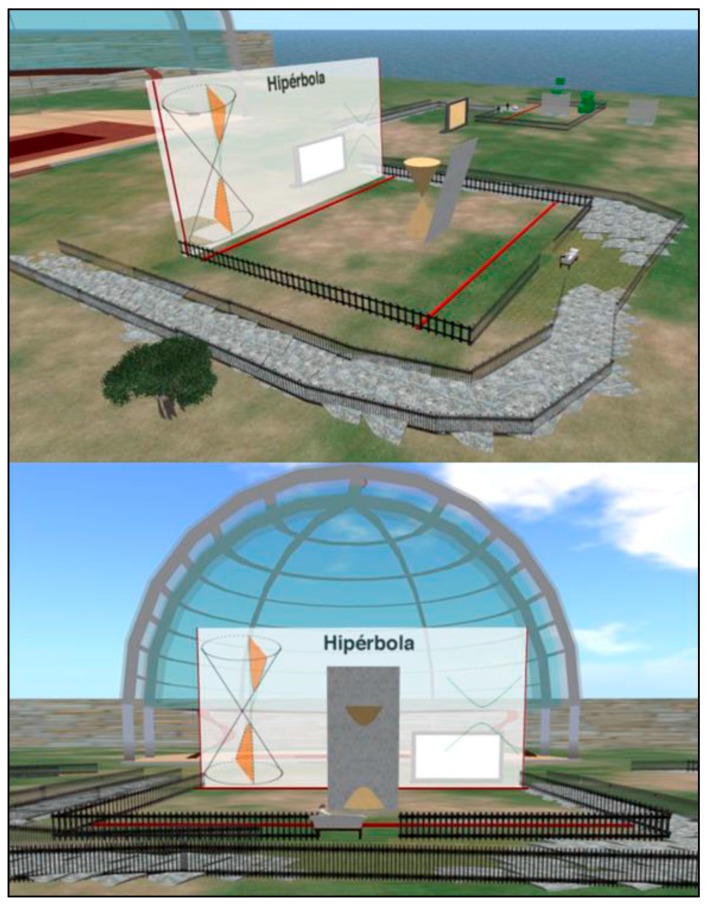
Two views of the virtual elements for teaching conic sections.

The student is able to modify the attributes of a virtual plane, such as the angle of tilt and shift. The conic section is generated as the intersection of this virtual plane and a cone, and is also portrayed in a virtual whiteboard, showing additional information about the conic section generated (see Figure 2).

#### 2.1.2. Tangible Interface Design

The FlyStick tangible interface emulates the way users interact with physical objects through their limbs. The tangible device was required to have the ability to perceive two types of muscle interactions: isometric and isotonic.

Isometric devices are also known as pressure devices because they use sensors that capture the force produced by muscle contractions where there is no significant change of position of the parts involved. These devices exert a force of equal magnitude in the opposite direction to the applied force.

Isotonic devices are also known as displacement devices because they use sensors that capture the movement of the human limb without exerting resistance. These devices provide freedom of movement because they do not exert any significant drag on the limb.

Research done by Zhai [49] suggests that it is better to use isotonic sensors when the speed and intuitiveness are the priority and to use isometric sensors when control and path quality is more important.

**Figure 3:**
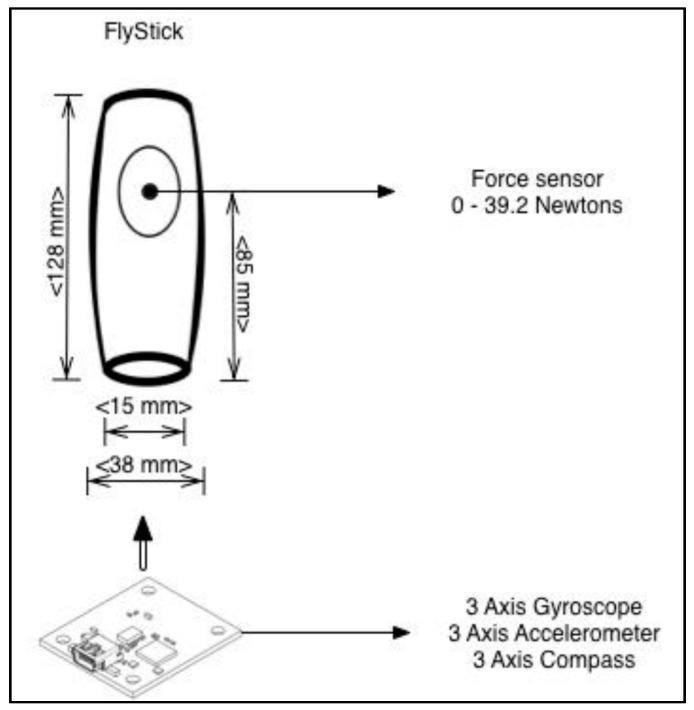
Schematic design of FlyStick.

The tangible interface prototype was designed to capture both types of muscle contractions through various embedded sensors, as shown in Figure 3. By combining the data obtained from a digital gyroscope, an accelerometer and a magnetometer, isotonic contractions are captured. On the other hand, a pressure analogical sensor captures the data to determine the amount of force that has been applied to the device (isometric contraction).

**Figure 4:**
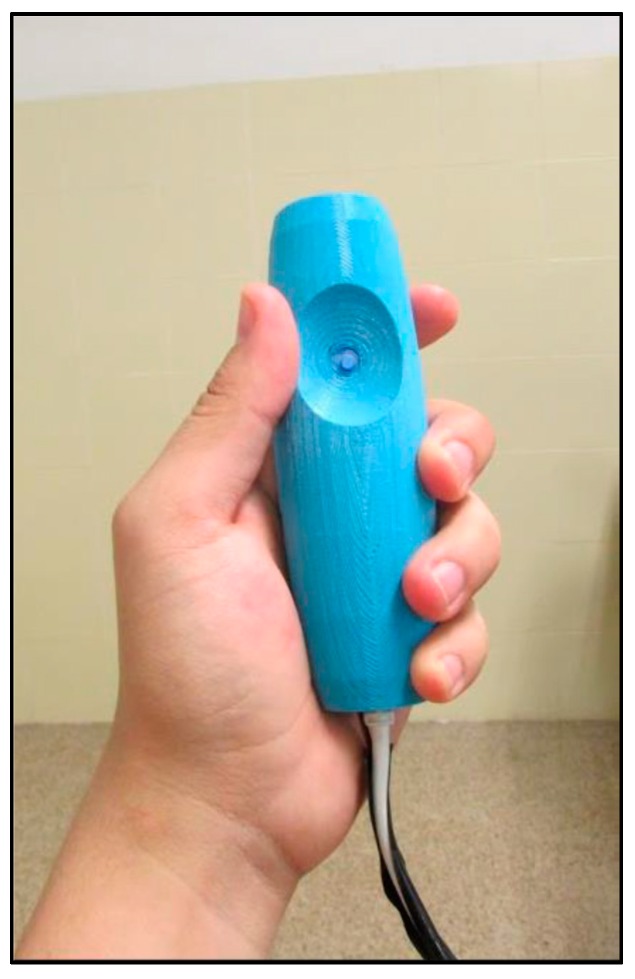
FlyStick.

Due to the nature of human interface devices, certain ergonomic aspects have been considered:

- *Shape and size*: The educational activity detailed below is aimed at students between 12 and 14 years old, so the device shape was designed taking into account the dimensions of other devices aimed at teenagers, such as Sony’s DualShock, whose shape and dimensions were used as inspiration to house the sensors on a single handed device (see Figure 4).
- *Material*: The selection of a material for the construction of the housing is a relevant aspect, since it is going to be on direct contact with the user’s skin. The chosen material was plastic PLA (polylactide), due to its characteristic of being organic and presenting no toxicity to humans.

The entire device was modelled using CAD tools and was later rendered and 3D printed using a 3D organic polymer printer.

### 2.2. PrimBox Tangible Interface

For the second tangible user interface, our proposal was to make a tangible interface for mixed reality that allowed the creation of new objects or figures in the virtual world in an intuitive way, thus solving some of the problems identified by Coban et al. [60]. In OpenSim virtual worlds (that follow the model of the well-known virtual world Second Life) the basic way of creating complex 3D structures (i.e., a house or a car) is by combining many basic geometrical figures (spheres, cubes, cones, etc., also called “prims”). This is done using the keyboard-and-mouse user interface as well as a series of menus. This is the process that is quite simplified by the tangible interface PrimBox. The tangible interface was to be used at a secondary education school, where students were to use the tool in tasks aimed to learn abstract knowledge.

#### Tangible Interface Design

Creating objects in virtual worlds is a complex task. The user does not perform natural movements when handling 3D elements using a keyboard-and-mouse interface. On the other hand, when the user employs a tangible interface for creating and editing objects in a virtual world, she is able to perceive a greater control over the object, since the movements performed are more natural, avoiding cognitive overload.

In this direction, a tangible interface was constructed to allow users to create and modify virtual objects, interacting directly with actual geometrical figures that represent geometrical objects in the virtual world. The user is able to change the attributes and characteristics of a geometrical object such as its size, position, orientation and color.

The requirements that the tangible interface had to meet were: (i) facilitating the insertion of a new geometrical figure in the virtual world; and (ii) facilitating the modification of the attributes (position, orientation, size and color) of geometrical figures in the virtual world, without requiring the user to manipulate a mouse or keyboard, in an easy and intuitive way.

A tangible interface, manufactured with health-safe materials, was designed for achieving these requirements. It consists of three elements: a container of geometrical figures, a reader of object attributes and a LCD display (used to offer additionally information), all of them embedded on a supporting board. Additionally, the set also includes two groups of tangible pieces (attribute cards and geometrical figures), which are the physical objects used to interact with the virtual world.

When the user wants to incorporate a new geometrical figure in the virtual world, she just selects the corresponding real-world plastic figure, and inserts it in the container of the tangible interface.

Once the figure has appeared in the virtual world, the user can modify its attributes using three orthogonal sliders that are attached to the sides of the container. The meaning of the sliders depends on what “card”, among a set of predefined cards, has been placed in a second container (card holder). For example, if the “size” card is located in the card holder, then the user can manipulate the sliders to change the size of the object in any axis. If the “position” card, or the “rotation” card, are used instead, the corresponding actions will be achieved with the same sliders. Finally, a fourth card for “color” can be used, and then the sliders represent the three color axes (RGB). The attributes changes take place immediately in the virtual world, and the concrete values of the changes are also shown on a LCD screen.

**Figure 5:**
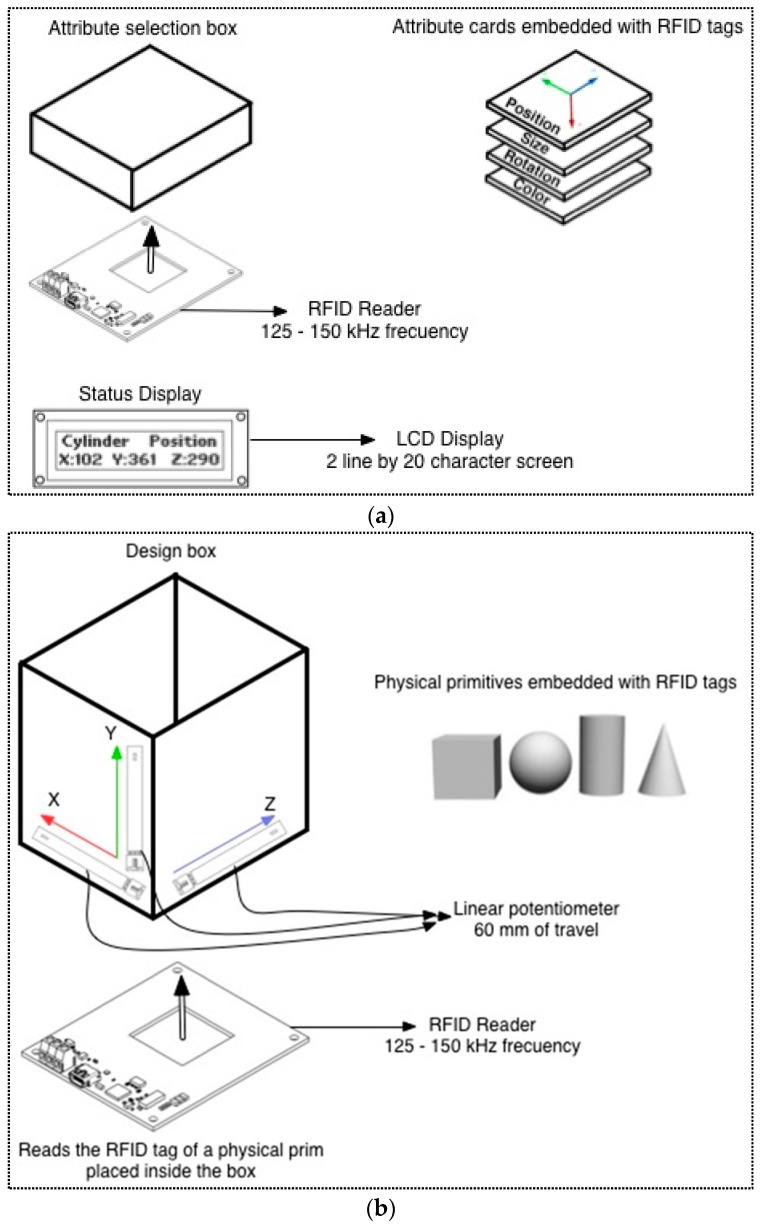
Design of Primbox. (**a**) Attribute selection box and attribute cards; (**b**) Desing box.

The container of figures incorporates an RFID reader (Figure 5) whose objective is taking information about the geometrical figure that has been inserted. This is done by an identifier (RFID tag) that is associated with each geometrical figure in the virtual world.

Three linear potentiometers are located at the edges of the container. Their displacement determines the value of the magnitudes of the attributes of the geometrical figure inserted in the virtual world. These attributes are: size, position, orientation and color. They are represented by cards, which contain an identifier (RFID tag). Reading these cards, it is done with another RFID reader prepared for this purpose.

The tangible interface features a LCD screen that displays in real time information such as the figure type that is being modelled, the attribute that has been selected and the values of the attributes that are changing.

In this case, the entire device was also modelled using CAD tools and was later rendered and 3D printed using a 3D organic polymer printer. This device was used in the same virtual world explained in the previous section, but with a different educational application, as will be explained below.

## 3. Implementing Educational Applications with the Tangible Interfaces: A Pilot Study

### 3.1. Research Design

The two tangible interfaces described above have been evaluated in “Florida Secundaria”, a high school in Valencia, Spain. The study was organized in four sessions of three hours. The first session was devoted to an introduction to virtual worlds. In this first session, students explored the virtual world, and learned how to create, move and rotate objects, how to interact with other avatars and how to customize the look of their avatar.

In the other three sessions the students performed several educational activities within the virtual world, where they used the tangible interfaces PrimBox and FlyStick.

The activities were aimed to learn geometry, with the main objective of developing spatial vision, taking advantage of the three-dimensional virtual world. In this study we settled a group that made the experiment using the mixed reality applications, and a control group that worked the same materials with a traditional methodology.

The traditional methodology consisted on explaining the geometrical concepts using the blackboard. Later, the students had to take some paper-and-pencil tests on the subject. In contrast to the traditional methodology, students using the tangible interfaces were practicing the geometrical concepts by themselves. The control groups worked in traditional classrooms, equipped with blackboards, where the students attended the explanations given by the teacher on the blackboard. The experimental group used a computer classroom, where the students interacted with tangible interfaces while exploring the virtual world.

In total 60 students participated in the study (30 students using mixed reality and 30 students using the traditional methodology).

For each group, the students were chosen randomly. After choosing the students we found that in the group using virtual worlds, 23% of the pupils had special educational needs, compared with 16% of pupils in the group using traditional methodologies. The special educational needs were mainly dyslexia, attention deficit disorder and hyperactivity (ADHD) and learning difficulties with some curricular adaptations.

The sessions were conducted on students of second and third year of secondary education (ESO). We prepared two activities (see Table 1). The activity 1 was performed in pairs. One student was given a sheet of paper showing a geometrical construction made with several (basic) geometrical figures. This student had to give appropriate instructions to her partner, who had to replicate the geometrical construction in the virtual world. The second student, who had not seen the sheet of paper, received all the necessary information from the first student using geometrical language (vertex, angles, points, half a point, position, rotation, etc.) The learning objective was to practice such technical language and the associated concepts.

Activity 2 consisted in learning how the different conic sections are generated from the intersection of a plane with a cone, by experimenting in the virtual world with a tangible interface.

These two activities were also performed by the control group, using a traditional methodology. In the case of the first activity, the second student had to draw the figure on paper based on the instructions received from the first student. In the case of the second activity the students attended a traditional (blackboard based) class where the teacher explained and draw the different conic sections on the blackboard.

Regarding the second activity, the student in the control group has a passive attitude where she hears the teacher’s explanations, while the student in the experimental group, besides listening to the teacher’s explanations, can interact with the tangible interface FlyStick and check how the conic sections are generated in real time. In order to evaluate the study, we have used:

- An introductory questionnaire about virtual worlds.
- A usability test about PrimBox and FlyStick.
- A semi-structured interview with the Mathematics teacher.
- Exercises and tests on the subject.

The questionnaire about previous knowledge on virtual worlds showed that most of the students were aware of virtual worlds and they had even played with them previously (see Table 2). That means that virtual worlds are attractive to students and their motivation is high: virtual worlds capture their attention and interest. Regarding ease of use, we found that virtual worlds were perceived as simple to use and to interact with, so the learning curve was very flat.

With respect to the usability questionnaire (see Table 3), students found the interaction with the tangible interfaces (PrimBox and FlyStick) simple and easy, as well as the completion of the proposed activities in the virtual world. The only problem found was the Internet connection, which sometimes was too slow.

After the study, we also made a semi-structured interview with the teacher of mathematics. A summary of the interview is presented in Table 4.

### 3.2. Activity 1: PrimBox

Activity 1, which used the tangible interface “PrimBox”, involved two student groups: the control group, which used a traditional teaching methodology, and the experimental group, which used the tangible interface and the virtual world. The activity was designed to develop the spatial perception and to practice the technical language of Mathematics and Geometry.

**Figure 6:**
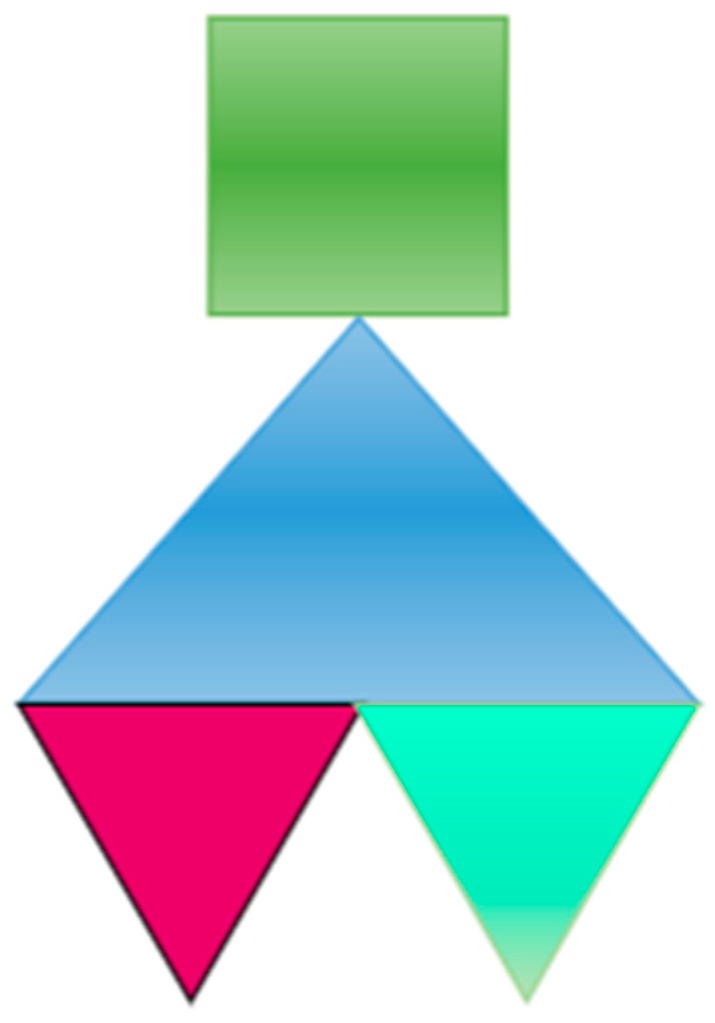
Example of composition of geometric figures for Activity 1.

In both groups, the activity was performed in pairs, where a student received a geometrical figure (see example in Figure 6) and had to explain, using a mathematical language, the position and orientation of each part of the figure, in order to copy it.

**Figure 7:**
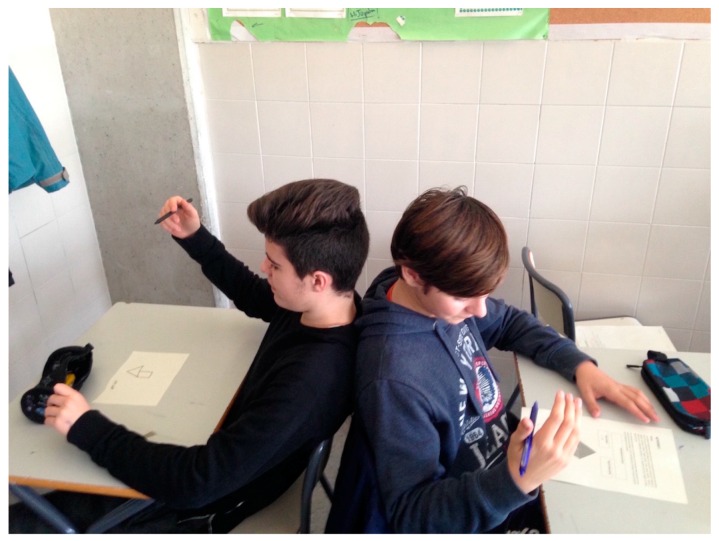
Students in the control group performing activity 1.

The control group was composed of 29 students of the 3th course of secondary education and the experimental group was composed of 30 students of the same course. In the case of the control group (see Figure 7), the activities were performed in conventional classroom where each pair of students interacted with each other in order to draw the required geometrical shape, one of them having seen the figure, and the other one having to draw it.

**Figure 8:**
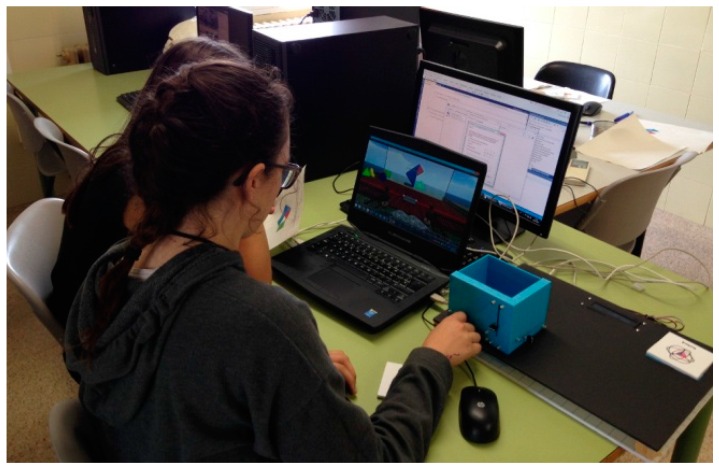
A student working on Activity 1 using the PrimBox tangible.

In the experimental group (see Figure 8), the activity was carried out in a computer classroom where the students used the virtual world to create objects, customized their avatars, explored the different activities that were present in the virtual island, and used the tangible interface PrimBox to perform Activity 1.

In order to evaluate the performance (in a 0–100 scale) three factors were taken into account: the appropriate use of the geometrical language, the correct orientation of the figure, and the correct positioning of the basic geometrical parts. We also used the same three metrics in the control group. From these three metrics, the teacher assessed a score for each pair of students in a 0–100 scale. The average score in this measurement was higher in the experimental group using the tangible PrimBox (all the students scored 100) while the control group got an average score of 74.5.

If we also take into account the time used to complete the task (less time means better performance) we can discriminate between students that got the same score. We may define an “efficiency” measure as the ratio between the quantities score and time in order to measure student performance. The larger is the score and the shorter is the time used, the larger is the efficiency. This measurement allows comparing the performance among students of the same group. However, for students in different groups the “time” quantity is not directly comparable. Students using the virtual world scenario may use some time in “auxiliary” activities (accuracy errors of the tangible interface, Internet connection failures, etc.) not directly related with the core of the activity. To allow a comparison of efficiency between the control and the experimental group, this time variable was normalized, dividing it by the average time in each group.

As can be seen in Table 5, the efficiency average in the experimental group using PrimBox (113.28 points) is slightly higher than the efficiency average of the control group (106.77 points), and also the standard deviation of the data is lower in the experimental group than in the control group. This means that the students who used PrimBox showed a higher efficiency compared to the students in the control group but this difference did not reach a level of statistical significance. In addition, we observed a higher motivation in students performing the activity using the tangible interface with respect to the students doing the activity using a traditional approach.

### 3.3. Activity 2: FlyStick

**Figure 9:**
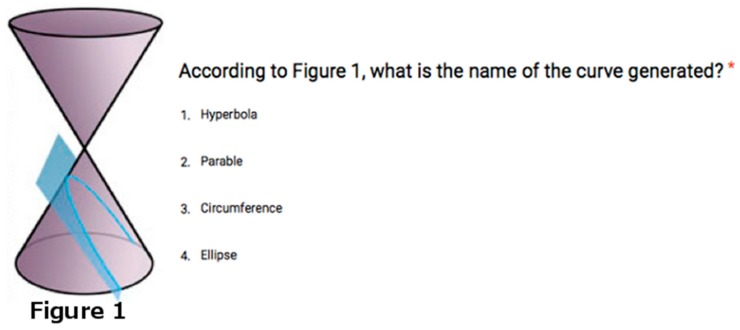
An example of question of the activity 2.

The second study aimed to improve the understanding of the conic curves. Two groups of students were involved in the study: one group using the tangible interface FlyStick and the other group using a traditional approach. In the traditional approach group students received an explanation of the conic curves on the blackboard. In the experimental group the students used virtual worlds for practicing the concepts using the tangible FlyStick. Later, both groups we were evaluated using a test where they were asked questions about the different conic sections (see an example of a question in Figure 9).

**Figure 10:**
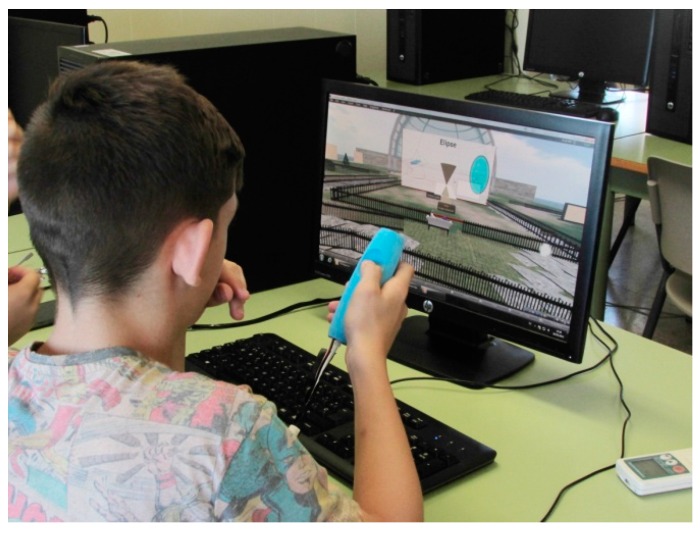
A student working on Activity 2 using the FlyStick tangible.

**Figure 11:**
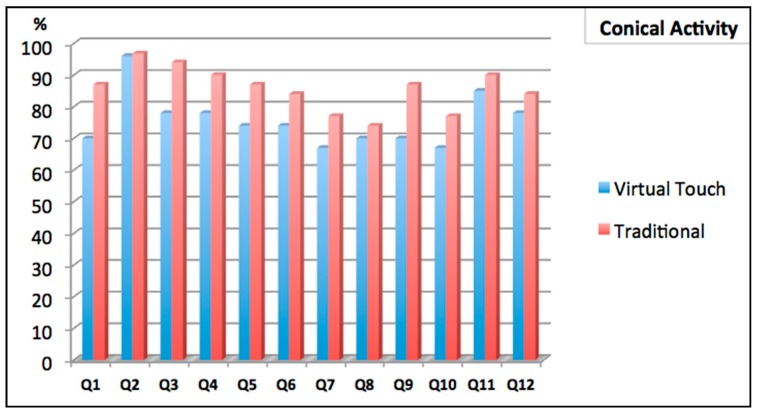
Comparative results: Virtual Touch and Traditional methodology (1st test).

**Figure 12:**
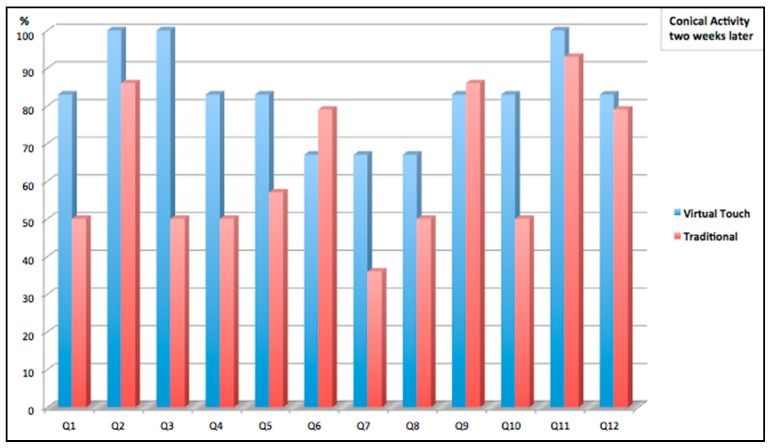
Comparative results: Virtual Touch and Traditional (two weeks later).

Two competence tests were used to assess learning (see Figure 10 and Figure 11). The first test took place just after the activity was performed (one group using FlyStick and the control group in a normal class). The second test took place two weeks later. Although the results of the first test were better for the students using the traditional methodology (see Figure 12), the students that had used FlyStick got better results in the second test: Virtual Touch methodology allowed better retention of the knowledge. This suggests that using Virtual Touch learning is more meaningful, probably because motivation makes students remember the concepts more accurately in a long term.

These activities took into account that students with special educational needs are more problematic when understanding an activity. However, students with special educational needs improved their results in the study with respect to their previous qualifications in Mathematics in an 86%. Doing activities with virtual worlds and tangible interfaces enabled them to improve mathematical competence, showing better performance.

As explained before, the students took two tests: one just after the activity was done, and a second one two weeks later. We observed that the students in the control group had significantly worse scores in the second test, while the students that used the mixed reality system got scores similar to their first scores. This fact suggest that the learning process has been more significant when using the mixed reality system.

To investigate for statistical significance we applied a paired *t*-test control as it is suitable for comparing ‘repeated measures’ (before and-after tests) for the same group of students. The control group was assessed at the time of the study and then control students repeated the test two weeks later. Similarly, for the experimental group, students took the test at the time of the study and then again two weeks later. In this way, we controlled for statistical significance between the initial test performance (during the study) and the performance two weeks later.

We checked for the two following hypotheses:

- H1: The scores for the students in the control group show no significant differences between the first and second tests.
- H2: The scores for the students in the mixed-reality group show no significant differences between the first and second tests.

The paired *t*-test control rejected null hypothesis H1 for the control group, and, therefore, there are significant differences between the scores in the two tests. In fact, 84.6% of these students got worse results in the test that they took two weeks later. On the other hand, the paired *t*-test failed to reject null hypothesis H2 and therefore, there were no significant differences in the scores that students in the mixed reality group obtained in these two tests. Therefore, the data support the conclusion that new knowledge is better retained when using the tangible interface. Using the mixed reality system, resulted to a more meaningful learning.

## 4. Conclusions and Future Work

From the study explained in this paper we can conclude that students were more motivated when using the Virtual Touch system for learning geometry, compared to the traditional approach. However, the novelty factor is probably having an impact. As future work, we need to evaluate Virtual Touch over a longer time span to check to what extent this “novelty” issue has an impact in the results.

Virtual Touch allows active learning because students are doing the activities by themselves; the teacher only gives them some initial instructions. Although students dedicated more time to do the exercises in the sessions using Virtual Touch, the system reduces the impulsivity of students to answer the questions and the students are more patient and relaxed while they are doing the activities. We observed that the students stayed one and half hour doing the activities in a good level of concentration, while it is quite difficult to maintain the interest and concentration so long using a traditional methodology. As future work, this has to be confirmed by repeating the experiment some more times.

Finally, the use of a virtual world platform instead of an ad-hoc 3D application will allow teachers to extend the educational applications, or even to create new ones by themselves. We have checked this feature in previous works [30]. One of the future areas of work will be to check that the system remains usable for teachers to develop their own educational applications when using the new tangible interfaces described in this paper. In Table 6 we present a summary of the strong and weak points identified.

## Figures and Tables

**Table 1:** Educational activities performed.

	Tangible Interface	Activity Description	Main Goal
**Activity 1**	VT PrimBox	One student is interacting with the virtual world constructing a geometrical figure following the oral instructions provided by a second student who is seeing a real world model	Using geometry language
**Activity 2**	VT FlyStick	The students, interacting with the tangible interface, generate various conic curves in the virtual world (circle, parabola, ellipse and hyperbola)	Understanding conic sections

**Table 2:** Result of the survey about previous knowledge on virtual worlds (26 students).

Kind of Question	Questions about Virtual Worlds (Session 1: Introduction to Virtual Worlds)	Yes	No
Previous Knowledge	Did you know virtual worlds previously?	17 (65.4%)	9 (34.6%)
	Have you ever played with virtual worlds before?	15 (57.7%)	11 (42.3%)
Easy to Use and Interactivity	Did you find it easy to interact in a virtual world?	25 (96.2%)	1 (3.8%)
	Do you think that it’s difficult changing the properties of objects in a virtual world?	10 (38.5%)	16 (61.5%)
	Have you found difficult to do collaborative activities in a virtual world?	4 (15.4%)	22 (84.6%)
Useful to Learn Mathematics and Geometry	Do you think that the virtual worlds help you to understand *X*, *Y*, *Z* coordinates?	19 (73.1%)	7 (26.9%)
	Do you think that virtual worlds can help you to learn Mathematics?	22 (84.6%)	4 (15.4%)
Motivation	Did you like the session about virtual worlds?	25 (96.2%)	1 (3.8%)
	Would you like to perform activities using virtual worlds in class?	25 (96.2%)	1 (3.8%)
	Would you like to perform activities using virtual worlds at home?	18 (69.2%)	8 (30.8%)

**Table 3:** Usability and user experience survey using Virtual Touch system (23 students).

Questions about Usability and User Experience (Session 2: Usability Survey)	Yes	No
Have you found the tangible elements easy to use?	8 (61.5%)	5 (38.5%)
Is Virtual Touch easy to use?	9 (69.2%)	4 (30.8%)
Was it quick to learn how to use the system?	11 (84.6%)	2 (15.4%)
Have you felt comfortable using Virtual Touch?	10 (76.9%)	3 (23.1%)
Virtual Touch facilitates group work or teamwork?	11 (84.6%)	2 (15.4%)
Have you needed help or assistance of the teacher?	2 (15.4%)	11 (84.6%)
Have the simulation of the activities in the virtual world been too complex?	5 (38.5%)	8 (61.5%)
Has the effort to solve the activities been very high?	1 (7.7%)	12 (92.4%)

**Table 4:** Semi-structured interview with the teacher.

Semi-Structured Interview Made to the Teacher	Answers
Have you ever played with virtual worlds before?	No
Have you found interesting the Virtual Touch system?	Yes
Have you needed technical support for using Virtual Touch?	Yes
Do you think that virtual worlds could improve the teaching of Mathematics?	Yes
	Virtual worlds can help to visualize scenarios on which subsequently apply mathematical processes.
	Virtual worlds can allow developing spatial vision of students.
	Virtual worlds are a creative and motivation environment.
	In a virtual world, students can share and collaborate on certain tasks in real time without worrying about the physical place where they are.
Did you find difficult to use the tangible?	No
Did you require a lot of effort to create training activities in the virtual world?	Yes
In your opinion, the major advantages of the Virtual Touch system are…	The tangible interface allowed recreating movements, which did not require great accuracy in the virtual world, in a more comfortable and closer to the student way.
In your opinion, the major disadvantages of the Virtual Touch system are…	Teachers need some training about the Virtual Touch system.
	It would be convenient to have a resource bank.
	The need for a complex infrastructure (server, ports, viewers…)
	The difficulty of designing activities that fit within the curriculum of the course.
Do you think the student may be distracted in the virtual world?	Yes
Rate the sessions	The students had a great willingness to perform activities
	In some activities students with special educational needs did as well as the other students.
	There should be a mechanism to assess the work of each avatar, like controlling the behavior of avatars.
Assessment of “Virtual Touch FlyStick”	Advantages: Ergonomic, lightweight, easy to install and use.
	Disadvantages: Improve the button to tilt the cutting plane (the button is too hard).
Assessment of “Virtual Touch PrimBox”	Advantages: easy to handle, easy to install and use.
	Disadvantages: The size of tangible. Couldn’t it be reduced?

**Table 5:** Results for Activity 1 using the PrimBox tangible.

Result	Control Group	Experimental Group
Sample size	n = 29	m = 30
Arithmetic average of results	X = 74.5	Y = 100
Arithmetic average on efficiency	X = 106.77	Y = 113.28
Variance	S_1_ = 5403.07	S_2_ = 1708.7
Standard deviation	SD_1_ = 73.50	SD_2_ = 41.33

**Table 6:** Strong and weak points identified.

Strong Points	Weak Points
Meaningful learning	Distraction in some students
Active learning	Need to have good equipment (high band width, computer with good graphic cards, configuration ports to connect with server.)
Reduce impulsivity	Evaluate the novelty factor in a longer period
Improve the motivation	Need to create and adapt all activities and teaching materials
	Need to perform training for teachers
	Difficulty controlling the behavior of avatars
